# A modality‐agnostic coronary artery habitat model for cardiac sparing in radiotherapy

**DOI:** 10.1002/mp.70595

**Published:** 2026-07-21

**Authors:** Chase Ruff, Nicholas Summerfield, Ming Dong, Prashant Nagpal, Adam Bayliss, Andrew M. Baschnagel, Carri K. Glide‐Hurst

**Affiliations:** ^1^ Department of Medical Physics University of Wisconsin‐Madison Madison Wisconsin USA; ^2^ Department of Radiation Medicine University of Wisconsin‐Madison Madison Wisconsin USA; ^3^ Department of Computer Science Wayne State University Detroit Michigan USA; ^4^ Department of Radiology University of Wisconsin‐Madison Madison Wisconsin USA

**Keywords:** cardiotoxicity, radiotherapy, segmentation

## Abstract

**Background:**

Emerging evidence suggests that the risk of cardiotoxicity increases with increased radiation dose to coronary arteries (CAs). However, robust tools to evaluate this increased burden for cancer patients are not available due to current limitations in imaging for radiotherapy treatment planning.

**Purpose:**

We have developed a novel, statistical methodology to define coronary artery “habitats”, or probabilistic, high‐risk regions to facilitate enhanced cardiac sparing in radiation therapy. Our modality‐agnostic approach leverages state‐of‐the‐art deep learning models and high‐resolution coronary CT angiography images for fully automatic prediction of CA habitats on varied radiation therapy datasets.

**Methods:**

Incorporating imaging data from two separate institutions and two cardiac segmentation challenge datasets, 182 coronary CT angiography (CCTA) and 60 thoracic radiotherapy volumes (simulation CT, 0.35T MRI) were evaluated. Images were labeled using nnU‐Net for CAs and whole‐heart and verified by a cardiovascular radiologist. Habitats were predicted by (1) template matching to select best‐fit CCTA, (2) deformably warping CAs from CCTA to each input image via deep learning models, (3) estimating each CA probability density function, and 4) final habitat derivation. Model parameters were optimized using a subset of 30 CCTA. Full‐branch (i.e., derived from all CA segments on CCTA) and main‐branch (i.e., derived from main CA branches only) habitats were predicted. Final habitats were evaluated on 60 CCTA, 34 simulation CT, and 26 MR‐Linac volumes via inclusion ratio and Hausdorff distance. Additional quantitative evaluation was performed with CAs with added planning organ‐at‐risk volumes (PRVs). Dosimetric correlation was evaluated between predicted habitats and CAs with added PRVs for 51 clinical treatment plans. To demonstrate applicability of our model to radiation treatment planning, habitat‐spared treatment plans were retrospectively re‐optimized for three patients and compared to re‐optimized plans sparing the whole‐heart.

**Results:**

Predicted full‐branch and main‐branch habitats contained 92.4 ± 8.9%–97.6 ± 4.5% and 89.7 ± 11.7%–98.2 ± 2.6% of CAs, respectively. Considering CAs with added PRVs, inclusion within habitats was lower compared to CAs alone, ranging from 83.1 ± 11.1%–92.3 ± 8.0% and 75.3 ± 15.0%–87.6 ± 9.4% for the CT‐SIM and MR‐linac datasets, respectively. When CAs were not contained within habitats, Hausdorff distance between main‐branch habitats and CAs ranged from 0.7 ± 1.6–4.4 ± 4.5 mm across all modalities. Predicted habitat volume was 2.1%–17.4% of the whole heart, on average. End‐to‐end prediction time was <4 min across all modalities. D_mean_ and D_0.03cc_ for habitats and CAs with an added PRV were strongly correlated (*r* >0.90) and in agreement (ICC >0.86) across 51 clinical plans for all CAs. CA D_0.03cc_ was reduced by up to 21.1 Gy in habitat‐spared plans, while D_mean_ was reduced by up to 18.5 Gy.

**Conclusions:**

Habitats contained on average >90% of CAs and 75%–92% of CAs with PRVs across evaluated modalities with strong agreement in dosimetric indices between manually delineated CAs and predicted main‐branch habitats. Substantial reductions in CA dosimetric endpoints were possible using habitat‐spared treatment plans while maintaining plan quality. Habitats serve as a clinically feasible alternative for CA delineation and enable treatment planning strategies to offer improved cardioprotection.

## 1 INTRODUCTION

**FIGURE 1:**
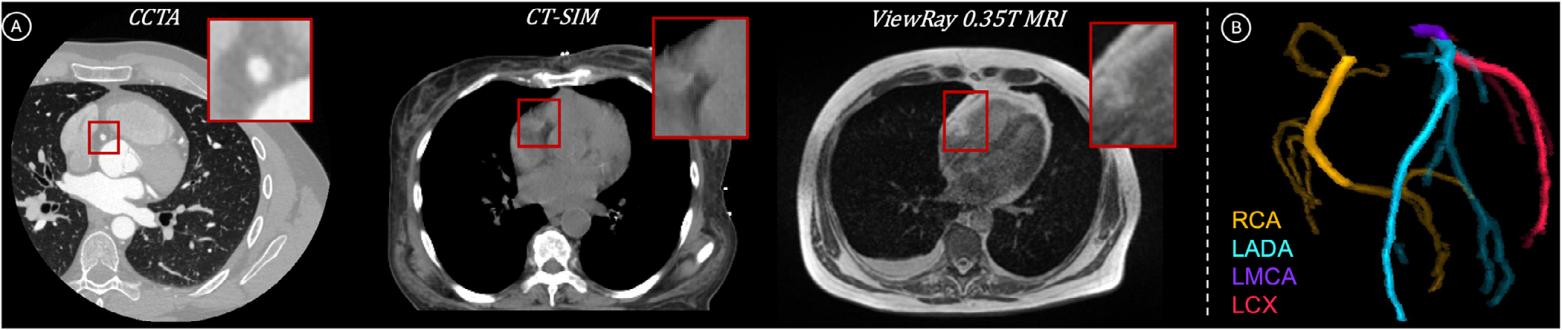
(a) Comparison between contrast‐enhanced CCTA (left), CT‐SIM (middle), and MR‐Linac (right), where the right coronary artery is highlighted with a red box. Coronary arteries are heavily blurred due to cardiac motion in the CT‐SIM/MR‐Linac datasets. (b) Example main‐branch coronary artery delineations based on current RT guidelines (bright) versus the full coronary artery branches delineated on CCTA (dark), where >50% of the right and left anterior descending coronary artery volumes are excluded, highlighting the advantages of an extended model for cardiotoxicity assessment.

Cardiotoxicity is a mounting problem in patients undergoing thoracic radiotherapy (RT).^1^ The coronary arteries (CAs) are especially radiosensitive, with recent studies revealing associations between increased dose to the right CA (RCA), left anterior descending CA (LADA), left main CA (LMCA), and left circumflex CA (LCX) with bradyarrhythmia, major adverse cardiac events (MACEs), ventricular tachycardia (VT), and atrial flutter.^3^ However, including CAs in treatment planning as organs‐at‐risk (OARs) is currently challenging. At present, the visualization of CAs on standard CT simulation scans (CT‐SIM) used in radiotherapy planning is limited by spatial and contrast resolution as well as the lack of cardiac gating features in conventional CT‐SIM platforms. When visible, only the main‐branch (i.e., proximal, medial, distal) CA segments can be delineated.^4^ Coronary CT angiography (CCTA) is increasingly considered the gold standard for CA imaging, allowing for visualization of the full CA tree.^5^, ^6^ Recent studies have demonstrated that inclusion of CCTA in treatment planning improves CA delineation to enable cardiac sparing.^7^ Yet, as of now, many RT departments lack electrocardiogram‐gated CT capabilities in their scanners required for CCTA acquisitions, thereby limiting opportunities for precise CA localization. Figure 1 highlights delineation of the CAs on CCTA compared to radiotherapy volumes (i.e., CT‐SIM, MR‐Linac). While the proximal CA segments have been shown to be highly radiosensitive,^8^ radiation dose to additional CA branches have been correlated with cardiotoxicities.^3^, ^9^, ^10^ For example, increased radiation dose to the first and second diagonal branches of the LADA have been associated with radiation‐induced stenosis.^9^ Additionally, the development of bradyarrhythmia from increased radiation dose to the nodal branches of the RCA has been suggested.^3^, ^11^ While correlating radiation dose to full‐branch CAs with downstream toxicities is currently challenged by radiotherapy imaging limitations, the dosimetric impact of dose to these smaller CA branches can be explored as advanced ECG‐gated^12^ and photon counting simulation CT^13^ technologies become increasingly available.

Due to the complexity and variability in CA anatomy, deep learning (DL)‐based CA segmentation solutions have been proposed.^14^, ^15^, ^16^, ^17^ However, current state‐of‐the‐art models still have low to moderate segmentation performance (Dice Similarity Coefficients (DSCs) ranging from 0.4–0.6 across CAs on CT‐SIM and 0.35T MR‐linac cohorts^15^, ^16^), underscoring the difficulty of CA delineation in conventional RT datasets. As an alternative to directly delineating the CAs, region‐based models have been proposed as OAR surrogates, such as manually defining high‐risk regions on CCTA,^18^ defining a sparing region based on the radial spread of CAs in relation to the aorta,^19^ or using a 1 cm thick arc along the heart wall to define a high‐risk zone.^20^ However, these simple models have only included the LADA or RCA and rely on manual definitions of regions representing the CAs. Dosimetric models have also explored defining a cardiac avoidance area (CAA, defined by the CAs, right atrium, superior vena cava, aortic valve root, and conduction nodes) based on image‐based data mining of delivered radiotherapy plans in lung cancer survivors, where increased dose to the CAA was associated with post‐treatment cardiotoxicities.^21^ However, these models are still dependent on manual delineation and group multiple substructures together, offering a straightforward model at the cost of simplifying substructure‐specific cardiotoxicities that may obscure important substructure/dose associations with clinical outcomes. Therefore, an accurate, multi‐modal approach is still needed to more precisely address CA radiation dose and associated toxicities in radiotherapy.

This work introduces and evaluates a novel, statistical framework for predicting CA “habitats” – probabilistic, high‐risk zones for each of the CAs—to facilitate cardiac‐spared treatment planning. Our modality‐agnostic approach leverages state‐of‐the‐art DL models and high‐resolution CCTA images for fully automatic prediction of CA habitats on varied RT‐relevant datasets. To allow for preferential sparing of smaller CA segments not including in standard radiotherapy definitions (i.e., LADA diagonal branches, RCA nodal branch), our ground‐truth CA definitions derived from CCTA include all visible artery branches. For comparison to ground‐truth CAs on CCTA where higher resolution data are available, the standard “full‐branch” model (based on the full, CCTA‐derived CA branches) is implemented. For comparison to manually delineated CAs on radiotherapy datasets, we use a “main‐branch” model (based on the proximal, medial, distal, and apical CA segments only). After validation of our model, we extend our model to retrospective radiotherapy treatment planning for three clinical thoracic cancer cases. Overall, we develop, validate, and extend our habitat model to treatment planning, with the overarching goal of delivering high precision cardiac sparing in radiotherapy.

## 2 MATERIALS AND METHODS

### 2.1 Data sources

A cohort of 393 diagnostic contrast‐enhanced CCTA scans acquired at our institution (0.49×0.49×0.63 mm^3^, reconstructed with a vendor‐provided DL algorithm (SnapShot Freeze 2, GE Healthcare) to minimize cardiac motion^22^) were identified from our institution using an IRB‐approved protocol. An additional 100 external CCTA cases from the “Multi‐Modality Whole‐Heart Segmentation”^23^ and “Automated Segmentation of Coronary Arteries”^17^ challenge datasets to facilitate automatic cardiac segmentation on diagnostic CCTA were analyzed. From the 493 available cases from our institution and the challenge datasets, a total of 182 scans containing patients with normal CA anatomy (e.g., no aneurisms, anomalous CA origin, or LCA dominance)^24^ were identified and used for model development. For model optimization, 30 (∼15%) cases were randomly chosen, and for evaluation, 60 (∼30%) CCTA cases^25^ were randomly selected from the total 182 CCTA. Overall, 182 CCTA were included for habitat model prediction, using 30 separate CCTA cases for optimization and 60 separate CCTA for model evaluation. Habitat model prediction was performed across the optimization and evaluation datasets using a leave‐one‐out approach, where for each CCTA, the remaining 181 CCTA cases are used to predict CA habitats.

To evaluate applicability to RT, two separate radiotherapy cohorts across two institutions were also included for model evaluation. Non‐contrast CT‐SIM images from a cohort of 34 patients with locally advanced non‐small cell lung cancer (NSCLC), and ViewRay 0.35T MR‐Linac volumes from 26 patients with thoracic and abdominal cancers, were labeled with the proximal, medial, and distal segments of the CAs following established RT atlases^4^ and cardiology guidelines.^5^ All CA delineations were reviewed and verified by a cardiovascular radiologist with >10 years of experience.

### 2.2 Coronary artery habitat prediction

**FIGURE 2:**
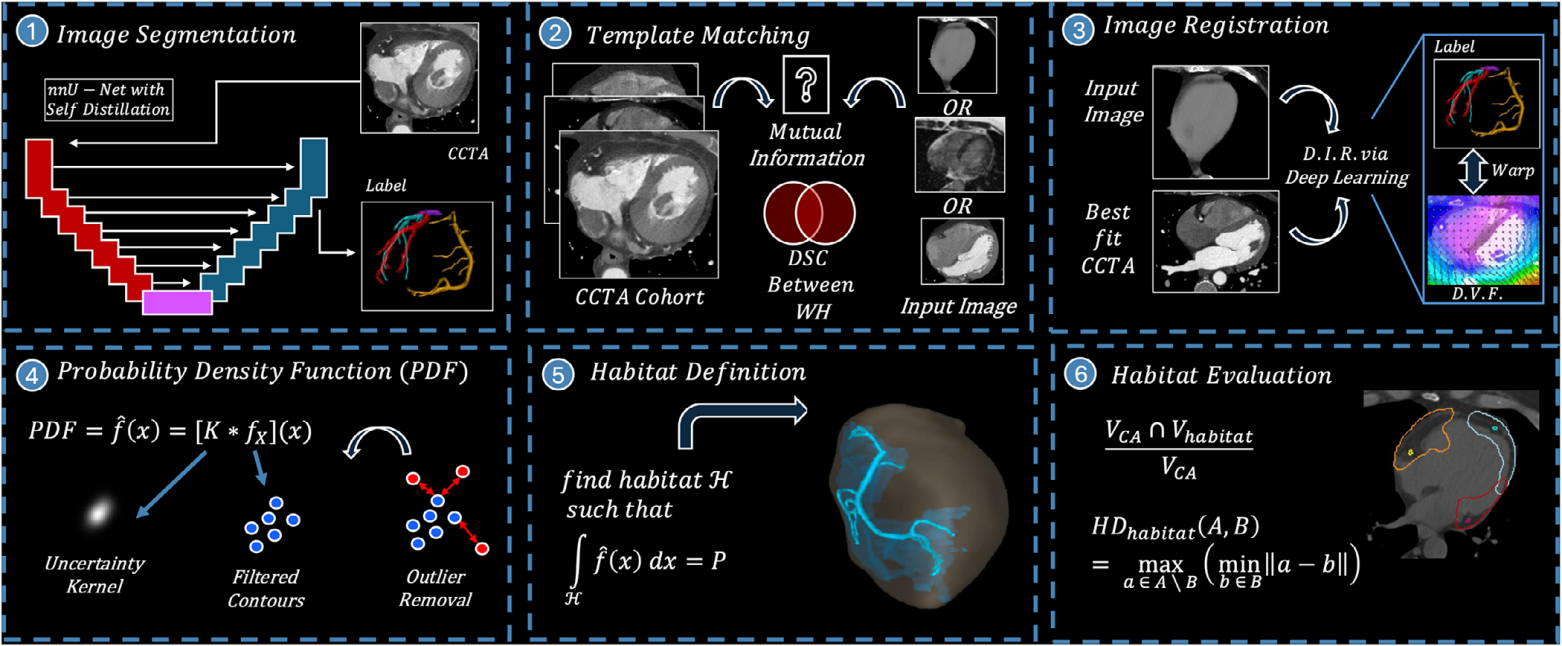
Habitat prediction workflow. (1) Deep learning CA segmentation on CCTA using nnU‐Net with self‐distillation. (2) Template matching based on the whole‐heart Dice Similarity Coefficient (DSC) and mutual information. (3) Deformable image registration between each best‐fit CCTA and the input image. (4) Probability density function (PDF) estimation via kernel density estimation, incorporating systematic and random uncertainties. (5) Habitats (H) are defined such that the integral of the PDF over the habitat is a given probability “P”. (6) Habitats are evaluated via inclusion ratio and Hausdorff distance.

Both main‐branch and full‐branch habitats for the RCA, LADA, LMCA, and LCX for a given input image were predicted using five steps as outlined in Figure 2, including: (1) automatic CA delineation on CCTA, (2) template matching, to determine the best‐fit CCTA cases, (3) deep‐learning based deformable image registration between the selected CCTA and input image, (4) estimation of the probability density function (PDF) for each coronary artery using kernel density estimation, and (5) prediction of the final artery habitats from the estimated PDF as described in detail below.

#### 2.2.1 Coronary artery segmentation on CCTA

Contours of the whole heart (WH), RCA, LADA, LMCA, and LCX were automatically delineated on CCTA using an in‐house nnU‐Net with dual self‐distillation framework,^15^, ^26^ with manual adjustment of predictions, including all visible CA branches in contour definitions^4^, ^5^ (Figure 2.1). Both the “main‐branch” (i.e., proximal, medial, distal, and apical branches) and “full‐branch” CAs were segmented. For the LMCA, there is only one artery segment, so the main‐branch and full‐branch delineations are equivalent.

#### 2.2.2 Template matching

Template matching is first performed via rigid registration between the input image and the CCTA cohort (Figure 2.2) to determine best‐fit CCTA cases, based on the DSC and Mutual Information Score (MI). The MI between each image is weighted by the WH DSC, selecting cases with a similar WH shape and size, as well as high correspondence between voxels in both images. The optimal number of best‑fit CCTA cases was determined through an iterative optimization process. Habitat predictions were generated on a fixed subset of 30 CCTA cases while varying the number of best‑fit cases used for model fitting from 5 to 30. At 15 best‑fit cases, further increases in the number of cases yielded less than a 1% improvement in CA‑habitat inclusion across all coronary arteries, indicating convergence of model performance. Therefore, 15 cases were selected as the optimal balance between model performance and computational efficiency (Figure S1).

#### 2.2.3 Registration

MultiGradICON,^27^ a DL‐based foundation model for multi‐modality deformable image registration (DIR, Figure 2.3), was used to register all CCTA to input images of any modality (i.e., CCTA, CT‐SIM, or MR‐Linac). The multiGradICON model was evaluated against additional DIR models via mean distance to agreement (MDA)^28^ for the task of inter‐patient, multi‐modal registration of cardiac/thoracic images, achieving the lowest MDA (i.e., highest accuracy, Table S1,2). All CA contours from best‐fit CCTA cases were deformably warped to the input image using multiGradICON and summed. Registrations were constrained to the WH contours, and the DSC between input and warped WH was used to assess registration quality, where values >0.90 were considered acceptable following AAPM Task Group Report No. 132.^28^

#### 2.2.4 Probability density function estimation

Following registration, the set of registered contours was filtered for outliers (>1 average nearest‐neighbor distance) and used to estimate the probability density function (PDF, Figure 2.4, Equation 1) of each underlying CA using kernel density estimation, where a kernel (K) is convolved with a distribution of data ($f_{X}$) to estimate the PDF ($\hat{f}\left(x\right)$).

(1) $$\mathrm{PDF}=\hat{f}\left(x\right)=\left[K*f_{X}\right]\left(x\right)$$

The convolution kernel shape/bandwidth are typically chosen to reflect the underlying uncertainty of the distribution. Systematic uncertainties due to deformable image registration, contour delineation,^28^, ^29^ and motion due to variable diastolic imaging windows^30^ are present for CCTA. For radiotherapy images, under the assumption of deep inspiration breath hold, additional uncertainties include cardiac motion^31^, ^32^ and inter‐fraction motion.^33^ Therefore, the uncertainties present on each imaging modality were represented with modality‐specific kernels, where errors are randomly sampled based on values from literature to form the kernels via Monte Carlo simulation.^34^, ^35^ All uncertainties besides cardiac motion were modeled with 3D Gaussian distributions, while cardiac motion was modeled with a skewed Gaussian in the left, anterior, and inferior directions, following the work of Shechter et al.^30^ Detailed values for each uncertainty estimate are provided in the Table S3.

#### 2.2.5 Habitat definition and post‐processing

Habitats were defined as the smallest region over which the estimated PDF integrates to a given probability, P (Figure 2.5, Equation 2).

(2) $$\;\int_{H}\hat{f}\left(x\right)dx=P$$

Based on typical definitions of PRVs,^36^ a value of 90% was used for *P*. Post‐processing of predicted habitats included filtering for voxels outside the largest connected region of the habitat and iterative Gaussian smoothing, where the Gaussian bandwidth was reduced until the smoothed habitat contained at least 95% of the voxels in the original habitat.

### 2.3 Habitat evaluation

#### 2.3.1 Quantitative evaluation

Following prior work defining high‐risk regions for the LADA by Lee et al.,^18^ final predicted habitats were evaluated (Figure 2.6) on CCTA and radiotherapy datasets via the inclusion ratio (Equation 3, a measure of the proportion of each CA encompassed by the prediction, where a higher value indicates a better prediction) and 95^th^‐percentile directional Hausdorff Distance (HD) between A, the excluded CA, and B, the corresponding predicted habitat (Equation 4, which characterizes the extent of spatial discrepancy between the CA and predicted habitat boundary, where a lower value indicates a better prediction). Only the main‐branch habitats were included for quantitative evaluation of radiotherapy datasets, due to limited visualization of smaller CA branches on these images.

(3) $$\mathrm{Incl}\mathrm{usion}\;\mathrm{Ratio}=\frac{V_{\mathrm{CA}}\cap V_{\mathrm{habi}\mathrm{tat}}}{V_{\mathrm{CA}}}$$

(4) $$HD_{habitat}\;\left(A,B\right)=\max_{a\in A\setminus B}\left(\min_{b\in B}∥a-b∥\right)$$

For evaluation of habitat size, the volume of each habitat as a percentage of the WH was calculated, along with the cross‐sectional area ratio between habitat and CA for the CT‐SIM cohort, following the methods in *Loap et al.* as a direct comparison.^20^ Quantitative metrics were assessed for statistically significant differences between imaging modalities using the Mann–Whitney U test (*α* = 0.05). The Wilcoxon signed‐rank test (*α* = 0.05) was used to test for statistically significant differences between the main‐branch and full‐branch habitats within the CCTA cohort and differences between individual CAs for all patients across all cohorts.

#### 2.3.2 Dosimetric validation

Before using predicted habitats as CA surrogates in treatment planning, a dosimetric comparison was performed between predicted habitats from the main‐branch model and delineated CAs on radiotherapy datasets. Planning OAR volumes (PRVs) are added to OAR structures to account for systematic and random errors throughout the course of treatment.^36^ For manually‐delineated CAs, artery‐specific margins of 4.6–8.5 mm (Table S4) were derived via the McKenzie‐van Herk formula (PRV=1.3·Σ+0.5·σ)^36^ using the same errors as when deriving PDFs. Derived PRVs were added to manually delineated CAs to reflect uncertainties incorporated into predicted habitats. For the radiotherapy datasets, additional quantitative comparisons were made between predicted habitats and manually delineated CAs with added PRVs to compare the two methods of incorporating uncertainties. From the 60 radiotherapy cases included in quantitative evaluation, 51 volumes had available RT dose structures and were used for dosimetric evaluation. Across the 51 clinical plans, D_0.03cc_ and D_mean_ were evaluated for the main‐branch habitats and CAs with added PRVs. To determine dosimetric agreement between predicted habitats and manually delineated CAs, the intraclass correlation coefficient (ICC) and Pearson coefficient (r) were calculated via linear regression for D_0.03cc_ and D_mean_, with a significance level of 0.05.

### 2.4 Example application: Habitat‐spared treatment planning

To demonstrate the use of habitats for cardiac‐spared treatment planning, three cases from the radiotherapy cohort with ultracentral targets abutting the heart^37^ and high CA dose (D_0.03cc_>25 Gy for RCA/LCX/LMCA, or V15Gy>10%^2^, ^3^, ^38^ for LADA) were selected for treatment plan re‐optimization. Clinical plans containing 2–4 VMAT arcs, where the contralateral lung was excluded from allowable beam angles, were re‐optimized using a fixed number of arcs and beam angles as in the original clinical plan. First, whole‐heart spared (WH‐spared) plans were made via a re‐optimization of clinically delivered plans and used for comparison. Two additional plans were created, sparing either (1) habitats or (2) ground‐truth CAs with added PRVs as OARs. D_0.03cc_ and D_mean_ constraints were placed on the main‐branch habitats/delineated CAs, while full‐branch habitats were included as dose fall‐off structures in optimization for the habitat‐spared plans. For both optimization and evaluation, in the case of overlap between CAs and the PTV, the residual with an added 5 mm margin (i.e., OAR−(PTV+5mm)) was used for CAs, CA PRVs, and predicted habitats, as target coverage was given a higher priority over cardiac sparing. Plan quality for all plans was assessed by a clinical medical physicist with advanced treatment planning expertise. CA D_mean_ and D_0.03cc_ were compared across the WH‐spared, CA‐spared, and habitat‐spared plans. To evaluate the potential sparing of the smaller CA branches not visible on radiotherapy imaging, D_mean_ and D_0.03cc_ to the full‐branch habitats were compared. Clinical OAR goals include D_mean_ <34 Gy and D_0.03cc_ <63 Gy for the esophagus, D_mean_ <30 Gy for the heart, D75% <5 Gy and D_mean_ <20 Gy for the lungs, and D_0.03cc_ <50 Gy for the spinal cord. Based on emerging evidence correlating cardiotoxicities with increased CA dose, clinical goals for the CAs include LADA V15Gy <10%,^2^ RCA D_0.03cc_ <25 Gy,^3^ LCX V15Gy <14%^2^, ^3^ and LMCA D_mean_ <27 Gy^2^.

## 3 RESULTS

### 3.1 Habitat model evaluation

**FIGURE 3:**
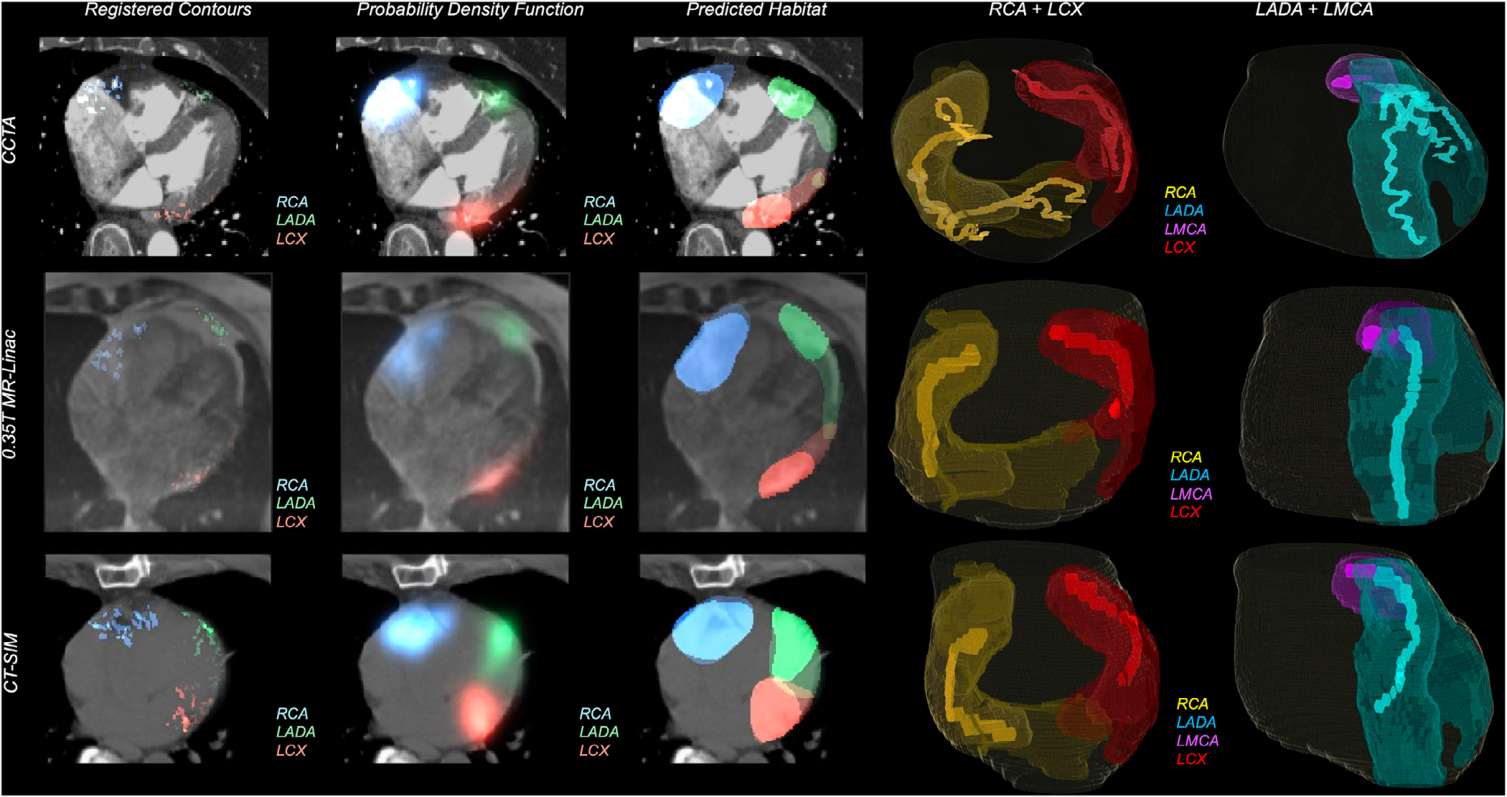
Registered contours, probability density functions, and predicted habitats (left) for example CCTA (top), 0.35T MR‐linac, (middle), and CT‐sim (bottom) cases. Volumetric renderings (right) of CAs overlaid with corresponding predicted habitats are shown for each case. Main‐branch habitats have brighter renderings while full‐branch habitats are represented by the larger, lightly shaded volumes shaded, larger volumes in the Predicted Habitats column in the middle and volumetric renderings for the RCA+LCX and LADA+LMCA. Acronyms defined in text.

**FIGURE 4:**
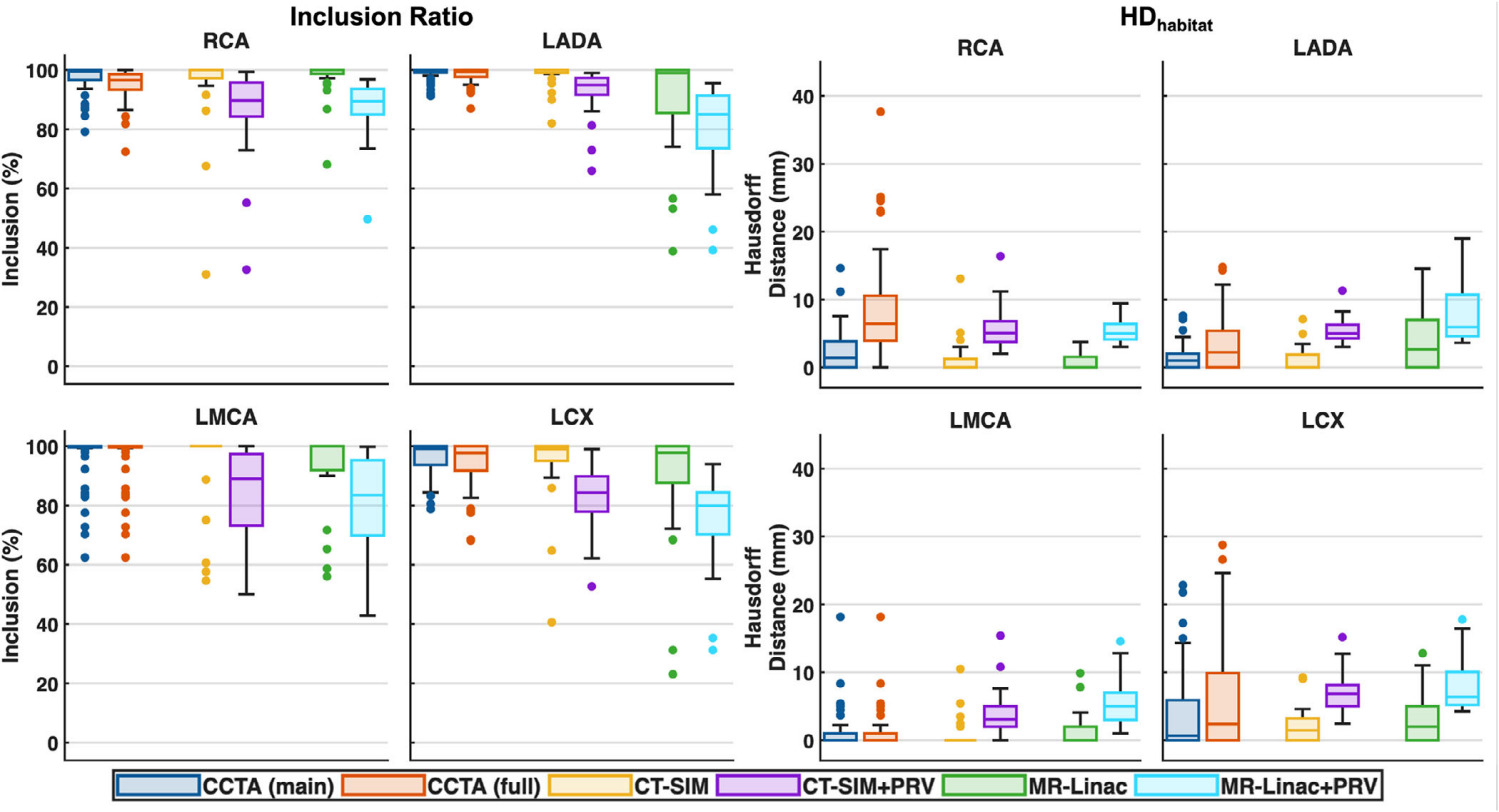
Quantitative evaluation for the main‐branch and full‐branch CCTA, CT‐SIM, and MR‐Linac cohorts, comparing inclusion ratio (left) and HD_habitat_ (right) for each coronary artery. Median and quartile values are shown with horizontal lines, with whiskers shown in black. Outliers are shown with solid circles, defined as data greater than 1.5·IQR away from the upper/lower quartiles.

Figure 3 highlights example habitat prediction steps (i.e., warped CAs, estimated PDF) and final predicted habitats are shown for selected CCTA, MR‐Linac, and CT‐SIM cases. Across all modalities, DSC for the WH between registered CCTA and input image was 97.5 ± 0.2% (range: 90.5%–99.5%). Inclusion ratio and HD_habitat_ for the CCTA, CT‐SIM, and MR‐Linac cohorts are shown in Figure 4. Full quantitative evaluation including end‐to‐end prediction time, habitat sizes as a percentage of the WH, HD_habitat_, and habitat‐CA inclusion across all cohorts are summarized in Table S5. Statistical testing results are displayed in Table S9,10. For main‐branch habitats, the average inclusion ratio across all CAs was >90%, ranging from 89.7 ± 11.7% for the LADA on MR‐Linac to 98.2 ± 2.6% for the LADA on CCTA. The largest HD_habitat_ (4.4  ±  4.5 mm) was for the LADA on the MR‐Linac cohort. Across all cohorts, main‐branch habitat size was largest for the RCA, followed by the LADA, LCX, and LMCA, ranging from 2.1%–11.4% of the WH volume (*p*<0.05). For the radiotherapy cohorts, additional comparisons were conducted between the delineated CAs, the CAs with estimated PRVs, and the predicted habitats using our framework. On average, inclusion within the habitats for CA+PRVs were 83.1 ± 11.1% to 92.3 ± 8.0% for the CT‐SIM and 75.3 ± 15.0% to 87.6 ± 9.4% for the MR‐linac datasets, which were all lower than the CAs alone. Examples of the lowest performing cases when evaluating CAs with added PRVs are highlighted in Supplementary Figure 2. For the CAs with PRVs, the average HD_habitat_ ranged from 3.8 mm (LMCA on CT‐SIM) to 8.2 mm for the LCX and LADA on MR‐Linac datasets. For the CCTA‐derived full‐branch habitats, the inclusion of full‐branch CAs ranged from 92.4 ± 8.9% to 97.6 ± 4.5%, while HD_habitat_ ranged from 1.4 mm to 9.9 mm and was lowest for the LCX and greatest for the RCA. For the RCA and LADA, inclusion ratio was lower (*p*<0.05) and HD_habitat_ was higher (*p*<0.05) for the full‐branch CAs compared to the main‐branch delineations for the CCTA cohort. Across all cohorts, the average end‐to‐end prediction time was <4 min.

### 3.2 Dosimetric validation

**FIGURE 5:**
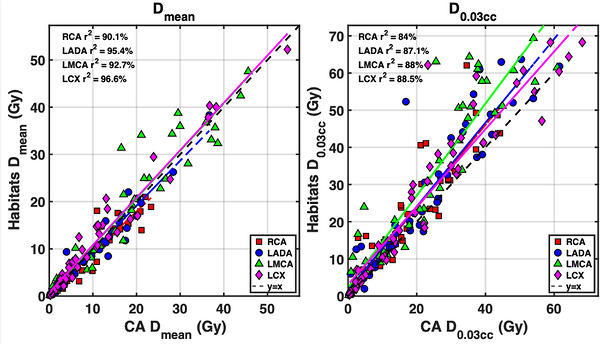
Comparison of D_mean_ (left) and D_0.03cc_ (right) for predicted habitats and ground‐truth CAs across 51 clinical treatment plans. Pearson coefficient (r) values (linear fit shown with solid line) are provided, with *y* = *x* plotted (dashed line, black) for comparison.

Figure 5 shows comparisons and linear regression results between manually delineated CAs with added PRV margins and predicted main‐branch habitats, for D_mean_ and D_0.03cc_. Across the evaluated 51 clinical plans, on average, the change in D_mean_ for predicted habitats with respect to manually delineated CAs with added PRVs was −0.8–0.5 Gy, while the change in D_0.03cc_ was 3.3 to 7.0 Gy. D_mean_ for manually delineated CAs and predicted habitats were significantly correlated for all CAs (*r* = 0.95–0.98, *p*<0.05). Similarly, D_0.03cc_ was significantly correlated between habitats and ground‐truth CAs (*r* = 0.92–0.94, *p*<0.05). Overall, between predicted CA habitats and manually delineated CAs, there was a strong, significant correlation (>0.9, *p*<0.05) for both D_mean_ and D_0.03cc_ for all arteries (Figure 5). For D_0.03cc_, ICC between habitats and manual CAs was lowest for the LMCA (0.86) and highest for the LCX (0.92), while for D_mean_, ICC was lowest for the RCA (0.94) and highest for the LCX (0.98). Tabulated ICCs and corresponding 95% confidence intervals are provided in Table S8.

### 3.3 Habitat‐spared treatment plans

**FIGURE 6:**
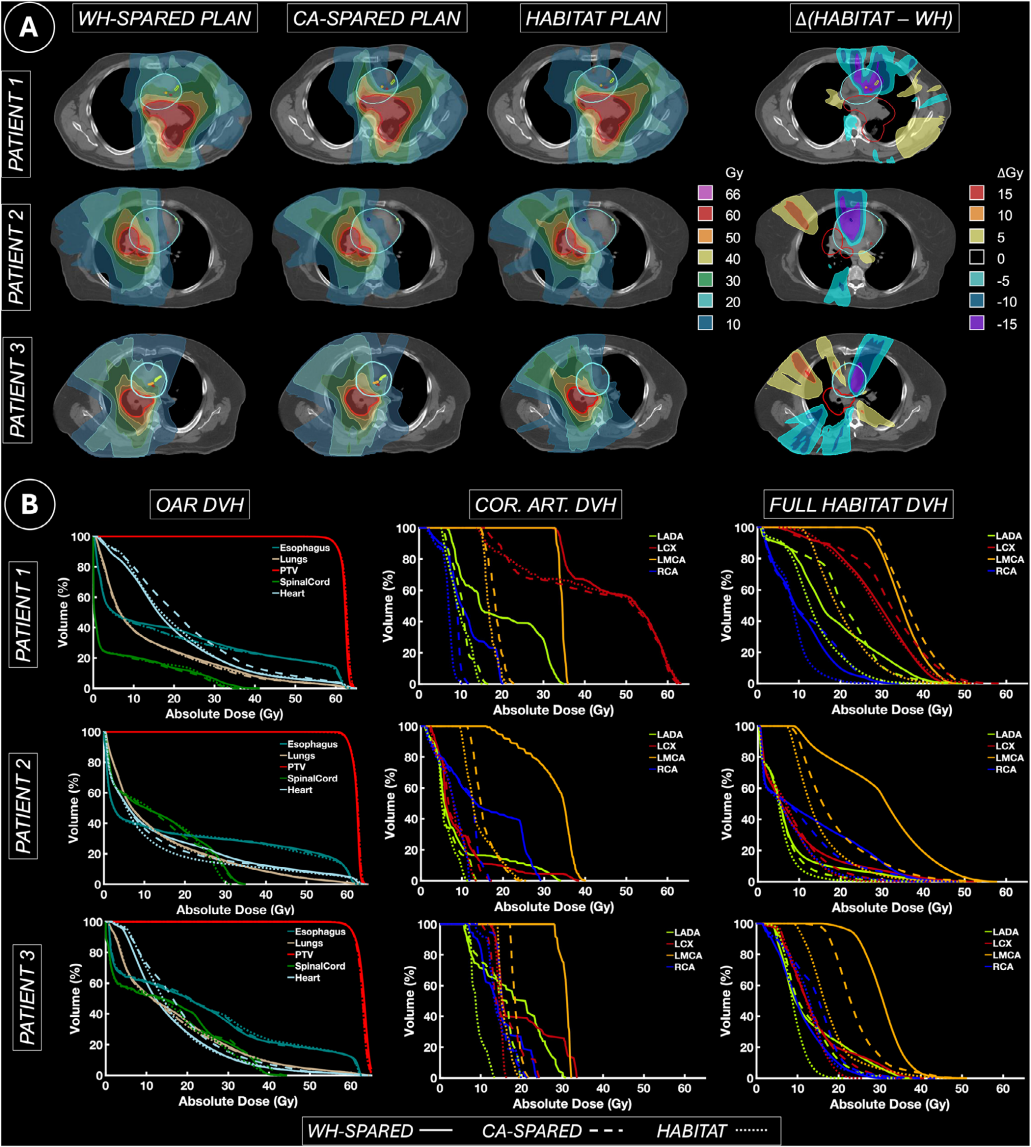
(a) Dose distributions for WH‐spared, CA‐spared, and habitat‐spared plans for 3 example patients. Dose difference maps for habitat‐spared and WH‐spared plans are shown (right). (b) DVHs for clinical OARs (left) coronary arteries (middle) and full‐branch habitat regions (right) are shown, for WH‐spared (solid), CA‐spared (dashed), and habitat‐spared (dotted) plans.

Example treatment plans (Figure 6A) and DVHs are shown (Figure 6B) for three selected patients. Dosimetric endpoints for all treatment plans are provided in the Table S6,7. Across habitat‐spared plans, D_0.03cc_ was reduced by up to 15.7, 21.1, 17.3, and 18.3 Gy for the RCA, LADA, LMCA, and LCX, respectively, compared to the WH‐spared plans. Similarly, D_mean_ was decreased between WH‐spared and habitat‐spared plans by 7.3, 9.7, 18.5, and 13.2 Gy. For all WH‐spared plans, LADA V15Gy exceeded 10%, which is associated with an increased risk of MACEs post‐RT.^2^ In all habitat‐spared plans, LADA V15Gy was reduced below 10%.

Comparing the CA‐spared and habitat‐spared plans, D_0.03cc_ was lower in the habitat‐spared plans, decreasing by up to 3.2, 4.0, 4.6, and 3.3 Gy for the RCA, LADA, LMCA, and LCX, respectively. Similarly, D_mean_ was reduced by up to 2.6, 2.2, 4.6, and 0.2 Gy. For the full‐branch habitats, which are estimations for the smaller CAs that are not visible on radiotherapy, differences between CA‐spared and habitat‐spared plans were much greater, with habitat‐spared plans reducing D_0.03cc_ and D_mean_ by up to 15.7 Gy and 5.8 Gy, respectively. All clinical goals met in the original plans were met in all reoptimized plans. Both habitat‐spared and CA‐spared plans were determined to be clinically acceptable by a clinical medical physicist with expertise in advanced treatment planning.

## 4 DISCUSSION

This work demonstrated the development, evaluation, and application of a CA habitat prediction model, using high‐resolution, diagnostic CCTA and state‐of‐the art DL methods, with the overarching goal of integrating habitats to enable cardiac‐spared treatment planning. End‐to‐end prediction time was ∼4 min per patient across imaging modalities and predicted habitats contained >90% of ground‐truth CAs on CCTA, CT‐SIM, and MR‐Linac MRI. Predicted main‐branch habitats contained >94% of CAs for CT‐SIM/CCTA and >90% of CAs for MR‐Linac. Generally, predicted habitats account for 2.1%–17.4% of the WH volume, although variations were observed across CAs and input modalities. Predicted habitats were the largest for the RCA (*p*<0.05), followed by the LCX (*p*<0.05), LADA (*p*<0.05), and LMCA (*p*<0.05). RCA dominance (i.e., a greater RCA volume compared to the left CAs) is prevalent across the general population,^24^ which corroborates with the larger volumes for the RCA habitats. Similarly, the LMCA is the smallest CA, defined as the most proximal 1.0–1.5 cm of the left CA,^4^, ^5^ in turn, yielding smaller predicted LMCA habitats. For the full‐branch habitats on CCTA, HD_habitat_ was larger on average than the main‐branch habitats due to the inclusion of smaller CA branches in contour definitions. For all CAs, this increase in HD_habitat_ was statistically significant (*p*<0.05). Since the LMCA only has one artery segment, there was no difference between the main‐branch and full‐branch delineations. For main‐branch habitats, HD_habitat_ was largest for the LADA across the MR‐Linac cohort (4.4 ± 4.5, *p*<0.05), potentially due to increased uncertainty from multi‐modality image registration. Across all modalities, HD_habitat_ was 4.4 mm at most and <2.4 mm on CT‐SIM, which is small compared to the inter‐observer error associated with manual CA delineation^39^ and Hausdorff distance of state‐of‐the‐art DL models,^14^, ^15^, ^16^ which often exceed 1 cm on CT‐SIM. Between predicted main‐branch habitats and manually delineated CAs, D_mean_ and D_0.03cc_ were both significantly correlated (*p* < 0.05) with *r* >0.9 for all CAs and ICCs ranging from 0.86 to 0.98, indicating strong dosimetric agreement.

Across all habitat‐spared plans, CA D_0.03cc_ and D_mean_ were reduced by up to 21.1 Gy and 18.5 Gy, respectively, compared to the WH‐spared plans (Figure 6, Table S7). Sparing predicted habitats resulted in lower CA dose compared to sparing the CAs directly, likely due to the increased size of the habitats. Additionally, full‐branch habitats were included in optimization, allowing for the potential sparing of smaller CA branches not visible through radiotherapy imaging (Figure 6). This is further demonstrated by the regions defined by full‐branch habitats, where D_0.03cc_ and D_mean_ were reduced by up to 15.7 Gy and 5.8 Gy, respectively, in habitat‐spared plans compared to CA‐spared plans. While these regions are not direct segmentations of the smaller, full‐branch CAs, the high inclusion of CAs in predicted habitats (>92% on average) on CCTA indicates that the full CA branches are likely contained within these estimated regions. In the habitat‐spared and CA‐spared treatment plans, target conformity was slightly reduced, and the streaking of lower doses (i.e., 10–30 Gy) was greater, with the 30 Gy and 40 Gy isodose lines extending into the chest wall in the habitat‐spared plans (Figure 6) while still meeting clinical treatment planning criteria. While these two trade‐offs are not clinically significant in a conventional 2 Gy fractionation scheme,^40^ the balance between sparing CA habitats and improving target conformity or reducing chest wall dose may be important in hypofractionated treatment schemes.^41^

An advantage to our model is that habitats are derived statistically from a population of diagnostic‐quality CCTA, instead of defined manually on radiotherapy images, resulting in fully automatic, data‐driven delineation of CA habitats. Another advantage of our model is its modality‐agnostic framework, allowing for prediction of CA habitats on common input modalities used in RT. While our preliminary work focused on CCTA, SIM‐CT, and MR‐Linac datasets, the habitat model framework could be extended to any additional thoracic imaging modalities in future work (i.e., dual energy CT, PET‐CT datasets, non‐contrast diagnostic CT). Other models have been proposed to generate high risk CA regions for treatment planning. *Lee et al.* developed a region‐based OAR surrogate for the LADA, where the LADA region (LADR) was first developed using CCTA, then manually delineated by four radiation oncologists on contrast‐enhanced CT‐SIM, achieving an inclusion ratio of 96%.^18^ Our habitat model achieves comparable inclusion on CCTA, with inclusion ratios for the LADA of 95.3% and 94.5% for main‐branch and full‐branch habitats, respectively. Similarly, *Loap et al.* developed an LADA surrogate—a high‐risk cardiac zone (HRCZ) – where a 1 cm‐thick arc, ranging from 1 to 8 cm in length, was delineated on the wall of the heart centered on the LADA.^20^ They reported a 27‐fold increase in HRCZ size compared to LADA size,^20^ which was smaller but comparable to the 38.2‐fold increase in size for our main‐branch habitat compared to the LADA for the CT‐SIM cohort. *Loap et al.* observed an over‐estimation of maximum dose to the HRCZ, which was similarly observed with predicted habitats compared to delineated CAs with an added PRV. Nonetheless, high *r* (>0.9) and ICC (>0.86) values indicate that D_0.03cc_ and D_mean_ strongly agree between our predicted habitats and ground‐truth CAs despite an observed over‐estimation of D_0.03cc_.

Despite an average inclusion ratio of >90% across all CAs and modalities, outliers were observed across both the CCTA and radiotherapy cohorts (Figure 4), with example cases highlighted in Supplementary Figure 2. The presence of pacemakers, abnormal cardiac anatomy (e.g., dextrocardia), or large tumors likely worsen registration error, contributing to decreased performance. Ultimately, a trade‐off exists between the number of best‐fit CCTA datasets used for habitat prediction, overall habitat size, and computational time. Additional best‐fit cases could be included to increase predicted habitat accuracy (Figure S1), which may subsequently increase habitat volume. If reduced computational time is desired, which may be valuable in an online adaptive scenario, an alternative approach would be to choose a template CCTA case based on population characteristics, predict CA habitats on the template case, and then deformably register the template and input image to warp predicted habitats to the input image. This approach would greatly reduce computation time while still allowing high‐resolution CCTA information to be transferred to radiotherapy images, at the cost of potential decreased accuracy due to lack of template matching and increased registration error from additional deformable image registration.^27^ Similarly, using fewer than 15 CCTA cases to predict habitats would reduce prediction time, at the potential cost of decreased accuracy.

An additional limitation of our work is the inherent introduction of inter‐patient, multi‐modality deformable image registration errors.^42^ However, in this work, registration error was minimized by constraining registrations to the heart volume and using a state‐of‐the‐art DL‐based algorithm (i.e., multiGradICON) which has been reported to have lower registration error compared to other comparator registration algorithms.^27^ While WH DSC is not a direct surrogate for the alignment of the CAs, the WH has been previously used to align images for motion quantification of cardiac substructures^43^, ^44^ and for registering multi‐modal imaging for diagnostic cardiovascular assessment.^45^ Additionally, registration error was accounted for in habitat prediction^27^ (Table S1), along with estimations of set‐up error^33^ and intra‐fraction motion^30^, ^32^ from the literature, which thus incorporates registration inaccuracies within the final predicted habitats. While uncertainty values will vary between patients, institutions, and treatment/image guidance techniques, these estimates included values derived from thoracic cancer cohorts across multiple institutions. Due to the flexibility of our habitat model framework, institution‐specific uncertainty values could be used to derive habitats, allowing for tailored and possibly more accurate predictions to local practices. Our habitat model framework relies on DL‐based image registration and GPU acceleration to improve accuracy and computational efficiency, respectively. If clinics lack the necessary dedicated GPUs, our framework can be implemented without GPU acceleration at the cost of reduced computational speed. Further, commercially available image registration algorithms could replace multiGradICON although performance should be validated before use.

The CAs with added PRVs demonstrated lower inclusion values within the habitats with higher HD_habitat_ than considering CAs alone (Figure 4). Potential reasons for this include the highly directional nature of cardiac motion for the CAs^32^ that is modeled within our habitat framework, while standard PRV definitions assume that intra‐fraction motion occurs independently in each anatomical direction, essentially treating the OAR as a rigid body.^36^, ^46^ To account for complex motion patterns and deformation, recent work by van Herk suggests that using a location‐dependent kernel may better account for highly directional random errors,^46^ which is a similar approach to our habitat model. Furthermore, while an error of 4 mm^4^ was assumed for radiotherapy volumes, prior work has shown that delineation errors can exceed 1.0 cm for the CAs.^47^ In cases with high delineation error, the smaller CA may still be contained within the habitat, while the addition of a PRV extends the OAR outside the predicted habitat, resulting in lower inclusion value. Finally, in instances of abnormal cardiac anatomy (i.e., dextrocardia, pacemakers) or local anatomical changes to the heart or nearby organs caused by the presence of large tumor volumes,^32^, ^48^ increased registration uncertainty may further contribute to the decreased performance. Nonetheless, HD_habitat_ for the CAs with added PRVs was on average 3.8 to 8.2 mm, indicating there is still high spatial correspondence between predicted habitats and CAs with added PRVs despite the observed reduction in model performance.

As advanced technologies become more accessible in RT clinics (e.g., photon counting CT, ECG‐gated simulation CT) and visualization of CAs on RT imaging is improved, our habitat model framework can be further validated against ground‐truth delineations of smaller CA branches which are currently visible only on CCTA. Full‐branch habitats could also be included in treatment planning studies including these advanced, high‐resolution RT images to evaluate the sparing of smaller branches via habitat‐spared planning. Further, correlational studies on delivered dose to full‐branch habitats and risk of cardiotoxicities (e.g., arrhythmias) can be explored. To evaluate dose to individual CA segments and smaller branches that currently cannot be resolved on RT imaging, segment‐level or branch‐level habitat model predictions could be implemented.

## 5 CONCLUSIONS

Predicted habitats were an accurate surrogate for manually delineated CAs, with high correlation of dosimetric endpoints between delineated CAs and predicted habitats. Application of our habitat model framework to radiotherapy treatment plan optimization yielded plans with reduced CA dose and acceptable plan quality. Future work includes incorporating other imaging modalities in habitat model prediction and assessing full‐branch habitats in thoracic cancer cohorts for deeper insights into RT‐related cardiotoxicities.

## CONFLICT OF INTEREST STATEMENT

Carri K. Glide‐Hurst reports research collaborations with Modus Medical, Inc, RaySearch, Leo Cancer Care, and Medscint, outside of the submitted work. Prashant Nagpal reports research collaborations with GE Healthcare, serves on advisory boards for Canon Medical and WCG Clinical, and owns personal stock in Moderna, outside the submitted work. Andrew M. Baschnagel reports research collaborations with the NIH and Wisconsin Partnership Program, outside the submitted work. Adam Bayliss, Ming Dong, Nicholas Summerfield, and Chase Ruff report no conflicts of interest.

## Supporting information




Supplementary Information

Supplementary Information

Supplementary Information

Supplementary Information

**Supplementary Information**cx

Supplementary Information

Supplementary Information

Supplementary Information

Supplementary Information

Supplementary Information

Supplementary Information

Supplementary Information
