# Supplementary figures and images for: A modality‐agnostic coronary artery habitat model for cardiac sparing in radiotherapy
Source: Med Phys. 2026 Jul 21;53(8):e70595. doi: 10.1002/mp.70595 (PMC13389350; doi:10.1002/mp.70595)

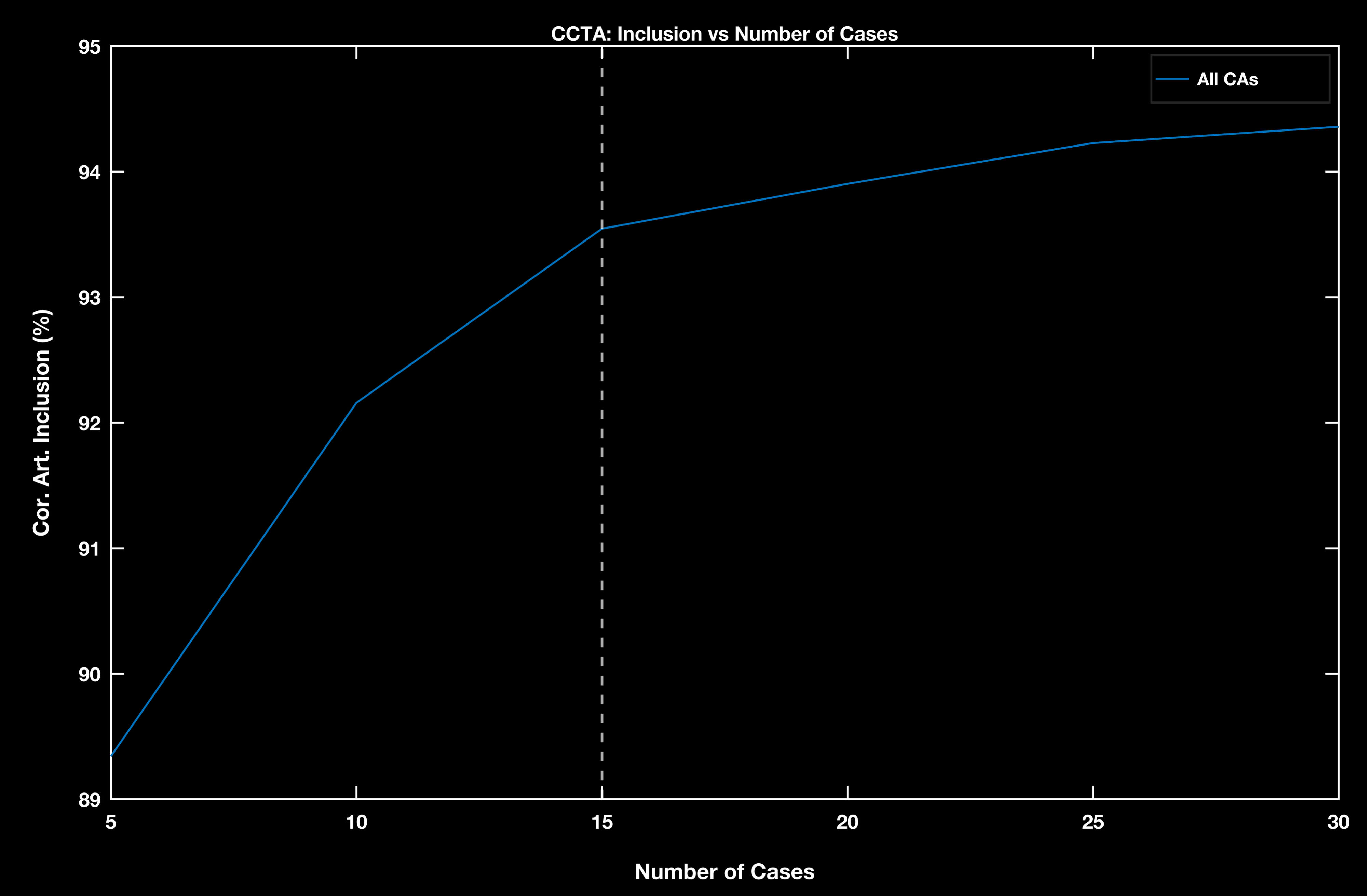

Supplement: Supplementary file 1 — Supplementary Information [file MP-53-0-s001.tiff]

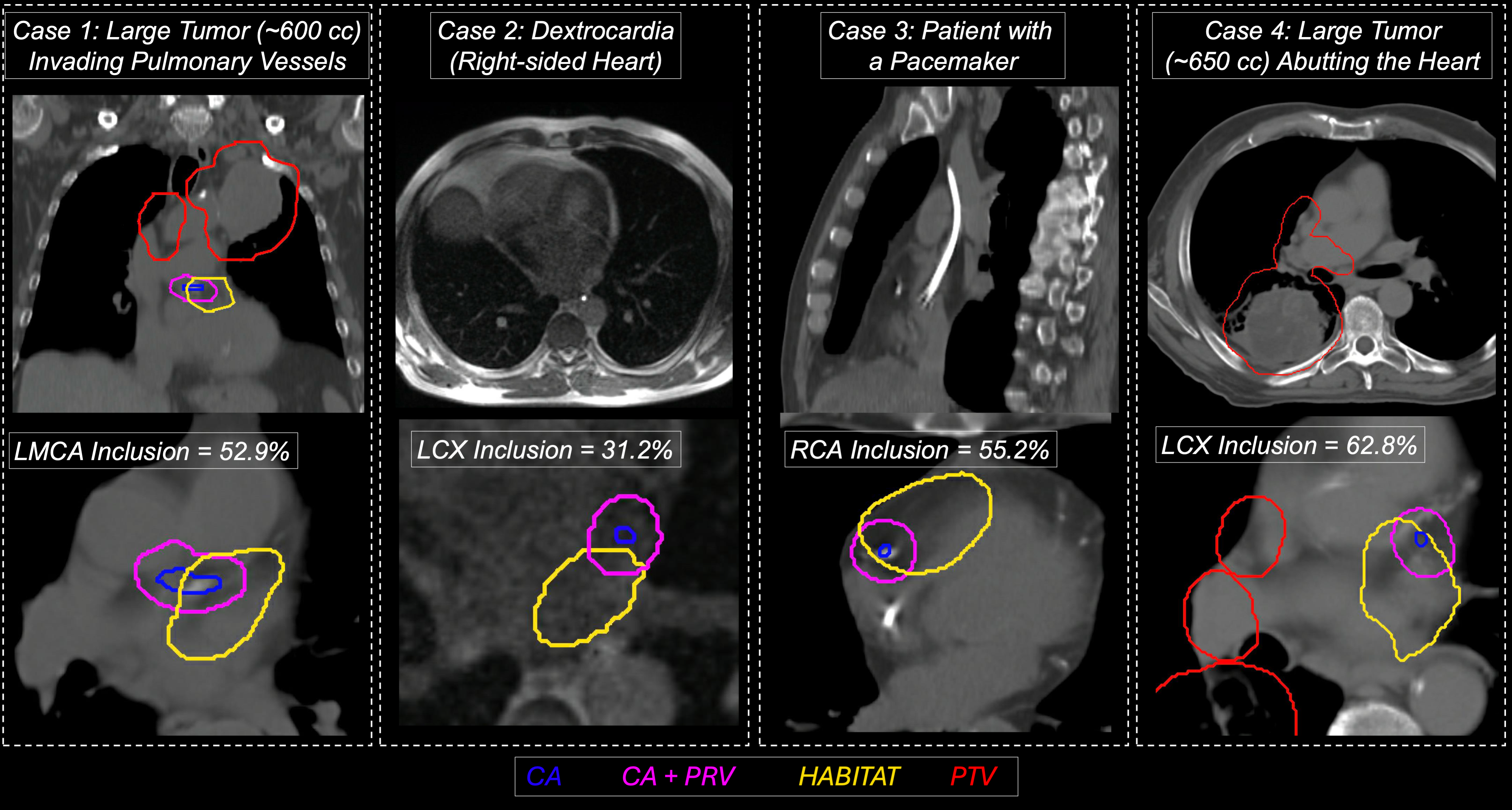

Supplement: Supplementary file 2 — Supplementary Information [file MP-53-0-s003.tiff]
