# Supplementary material for: A modality‐agnostic coronary artery habitat model for cardiac sparing in radiotherapy
Source: Med Phys. 2026 Jul 21;53(8):e70595. doi: 10.1002/mp.70595 (PMC13389350; doi:10.1002/mp.70595)
Supplement: Supplementary file 3 — Supplementary Information [file MP-53-0-s004.docx]

Supplementary Table 1: Evaluation of multiGradICON Registrations

| **MULTIGRADICON** | **CCTA/CCTA (n=66)** | **CCTA/TPCT (n=60)** | **CCTA/MR-LINAC (n=60)** |
| --- | --- | --- | --- |
| **SUBSTRUCTURE** | MDA (mm) | MDA (mm) | MDA (mm) |
| LEFT VENTRICLE | 3.2 (1.4) | 4.0 (1.3) | 3.6 (1.2) |
| RIGHT VENTRICLE | 4.7 (2.8) | 5.5 (1.8) | 6.8 (2.6) |
| LEFT ATRIUM | 2.3 (0.9) | 3.8 (0.8) | 4.1 (1.1) |
| RIGHT ATRIUM | 4.1 (1.5) | 6.3 (2.3) | 7.6 (3.6) |
| ASCENDING AORTA | 2.9 (2.8) | 5.8 (3.0) | 6.2 (3.1) |
| AVN | 4.5 (1.9) | 6.4 (3.2) | 5.1 (2.6) |
| AVERAGE | 3.6 (2.2) | 5.3 (2.5) | 5.5 (2.9) |

| FINAL REGISTRATION UNCERTAINTIES | CCTA | TPCT | MR-LINAC |
| --- | --- | --- | --- |
|  | 3.6 mm | 5.3 mm | 5.5 mm |
