# Supplementary material for: A modality‐agnostic coronary artery habitat model for cardiac sparing in radiotherapy
Source: Med Phys. 2026 Jul 21;53(8):e70595. doi: 10.1002/mp.70595 (PMC13389350; doi:10.1002/mp.70595)
Supplement: Supplementary file 4 — Supplementary Information [file MP-53-0-s010.docx]

Supplementary Table 2: Comparison to Additional Registration Models

| **DEMONS** | **CCTA/CCTA (n=66)** | **CCTA/TPCT (n=60)** | **CCTA/MR-LINAC (n=60)** |
| --- | --- | --- | --- |
| **SUBSTRUCTURE** | MDA (mm) | MDA (mm) | MDA (mm) |
| LEFT VENTRICLE | 7.2 (2.5) | 8.8 (3.3) | 9.8 (4.2) |
| RIGHT VENTRICLE | 7.6 (2.3) | 9.2 (3.1) | 12.3 (4.5) |
| LEFT ATRIUM | 7.0 (2.4) | 7.7 (2.3) | 11.7 (3.6) |
| RIGHT ATRIUM | 7.4 (2.1) | 11.1 (3.8) | 15.4 (4.8) |
| ASCENDING AORTA | 7.1 (3.1) | 8.1 (3.7) | 10.8 (3.9) |
| AVN | 5.9 (2.7) | 6.7 (2.6) | 7.8 (3.5) |
| AVERAGE | 7.0 (2.6) | 8.6 (3.5) | 11.3 (4.7) |
| **UNIGRADICON** | **CCTA/CCTA (n=66)** | **CCTA/TPCT (n=60)** | **CCTA/MR-LINAC (n=60)** |
| **SUBSTRUCTURE** | MDA (mm) | MDA (mm) | MDA (mm) |
| LEFT VENTRICLE | 3.0 (1.2) | 6.7 (3.4) | 4.1 (1.7) |
| RIGHT VENTRICLE | 4.3 (2.3) | 6.0 (2.3) | 7.9 (2.8) |
| LEFT ATRIUM | 1.9 (0.8) | 4.2 (1.3) | 4.4 (1.2) |
| RIGHT ATRIUM | 3.9 (1.5) | 6.8 (2.4) | 10.0 (4.1) |
| ASCENDING AORTA | 2.1 (2.4) | 6.4 (4.6) | 8.3 (3.6) |
| AVN | 4.3 (2.1) | 5.7 (2.8) | 5.5 (2.4) |
| AVERAGE | 3.3 (2.0) | 6.0 (3.1) | 6.7 (3.6) |
