# Supplementary material for: A modality‐agnostic coronary artery habitat model for cardiac sparing in radiotherapy
Source: Med Phys. 2026 Jul 21;53(8):e70595. doi: 10.1002/mp.70595 (PMC13389350; doi:10.1002/mp.70595)
Supplement: Supplementary file 5 — Supplementary Informationcx [file MP-53-0-s005.docx]

Supplementary Table 3: Overview of systematic and random errors used for deriving habitat kernels and planning organ-at-risk margins. *Estimated in Supplementary Table 1

|  | Source of Uncertainty | Value (mm) | | Uncertainty Distribution |
| --- | --- | --- | --- | --- |
|  |  | CCTA | Radiotherapy |  |
| Systematic Errors | Image Registration | 3.6* | 5.5* | Gaussian |
|  | Contour Delineation | 2.0 | 4.0 | Gaussian |
|  | Cardiac Gating Timing (CCTA only) | 1.0 | N/A | Gaussian |
|  | Average Inter-fraction Motion | N/A | 1.9 – 2.9 | Gaussian |
| Random Errors | Cardiac Motion | N/A | 4.3 – 11.0 | Skewed Gaussian |
|  | Standard Deviation of Inter-fraction Motion | N/A | 2.0 – 3.4 | Gaussian |
