# Supplementary material for: A modality‐agnostic coronary artery habitat model for cardiac sparing in radiotherapy
Source: Med Phys. 2026 Jul 21;53(8):e70595. doi: 10.1002/mp.70595 (PMC13389350; doi:10.1002/mp.70595)
Supplement: Supplementary file 6 — Supplementary Information [file MP-53-0-s009.docx]

Supplementary Table 4: Planning Organ-at-risk Volumes (PRVs) for Manually Delineated Coronary Arteries

| Coronary Artery | PRV (mm) | | |
| --- | --- | --- | --- |
|  | A-P | R-L | S-I |
| RCA | 7.1 | 8.5 | 6.0 |
| LADA | 5.3 | 5.6 | 7.4 |
| LMCA | 5.2 | 4.7 | 4.6 |
| LCX | 5.9 | 6.5 | 6.0 |
