# Supplementary material for: A modality‐agnostic coronary artery habitat model for cardiac sparing in radiotherapy
Source: Med Phys. 2026 Jul 21;53(8):e70595. doi: 10.1002/mp.70595 (PMC13389350; doi:10.1002/mp.70595)
Supplement: Supplementary file 7 — Supplementary Information [file MP-53-0-s007.docx]

Supplementary Table 5: Quantitative evaluation of main-branch and full-branch habitats, for CCTA, CT-SIM, and MR-Linac datasets. For CT-SIM and MR-Linac predictions, added PRV margins were delineated following the McKenzie-van Herk formalism^1^. CCTA predictions are evaluated against CA delineations with no added PRV.

|  | **Modality** | **CCTA (n=60)** | | **CT-SIM (n=34)** | | **MR-Linac (n=26)** | |
| --- | --- | --- | --- | --- | --- | --- | --- |
|  |  | **Main-Branch** | **Full-Branch** | **CAs** | **CAs+PRV** | **CAs** | **CAs+PRV** |
|  | **Time (mm:ss)** | 3:51 ± 0:11  (3:19, 4:06) | | 3:50 ± 0:10  (3:29, 4:04) | | 3:43 ± 0:11  (3:16, 4:00) | |
| **CA Volume (% of Heart)** | **RCA** | 9.8 ± 1.4  (6.7, 13.1) | 17.4 ± 1.9  (13.3, 21.0) | 11.5 ± 2.1  (9.6, 12.3) | | 11.3 ± 2.0  (9.0, 13.4) | |
|  | **LADA** | 8.4 ± 1.3  (5.6, 12.4) | 11.9 ± 1.4  (8.5, 15.1) | 9.2 ± 1.2  (6.7, 11.0) | | 8.6 ± 1.0  (6.2, 10.3) | |
|  | **LMCA** | 2.1 ± 0.4  (1.6, 3.2) | 2.1 ± 0.4  (1.6, 3.2) | 2.5 ± 0.4  (2.0, 3.3) | | 2.8 ± 0.3  (2.1, 3.5) | |
|  | **LCX** | 6.3 ± 0.7  (4.7, 8.1) | 10.6 ± 1.2  (8.2, 12.9) | 6.8 ± 1.0  (5.4, 8.1) | | 6.5 ± 0.7  (5.2, 7.5) | |
| ***HD_habitat_* (mm)** | **RCA** | 2.3 ± 2.8  (0.0, 14.6) | 8.7 ± 7.4  (0.0, 37.6) | 1.2 ± 2.8  (0.0, 13.1) | 5.7 ± 2.8  (2.0, 16.4) | 0.4 ± 1.1  (0.0, 3.7) | 5.4 ± 1.7  (3.0, 9.4) |
|  | **LADA** | 1.4 ± 1.9  (0.0, 7.6) | 3.6 ± 3.9  (0.0, 14.8) | 1.1 ± 1.7  (0.0, 7.1) | 5.5 ± 1.8  (3.0, 11.4) | 4.4 ± 4.5  (0.0, 14.6) | 8.2 ± 4.4  (3.6, 19.0) |
|  | **LMCA** | 1.0 ± 2.8  (0.0, 18.1) | 1.0 ± 2.8  (0.0, 18.1) | 0.7 ± 1.6  (0.0, 10.5) | 3.8 ± 3.1  (0.0, 15.4) | 1.5 ± 2.8  (0.0, 9.8) | 5.4 ± 3.1  (1.0, 14.6) |
|  | **LCX** | 3.9 ± 5.7  (0.0, 22.8) | 6.2 ± 7.4  (0.0, 28.7) | 2.4 ± 3.2  (0.0, 9.3) | 7.0 ± 2.6  (2.4, 15.1) | 3.3 ± 3.3  (0.0, 12.8) | 8.2 ± 3.7  (4.2, 17.8) |
| **Inclusion (% of CA within habitat)** | **RCA** | 97.4 ± 4.4  (79.2, 100) | 95.0 ± 5.1  (72.5, 100) | 95.3 ± 14.0  (31.1, 100) | 87.1 ± 13.4  (32.7, 99.3) | 97.6 ± 10.3  (68.2, 100) | 87.6 ± 9.4  (49.7, 96.8) |
|  | **LADA** | 98.8 ± 2.2  (91.3, 100) | 98.2 ± 2.6  (86.9, 100) | 95.3 ± 8.9  (82.1, 100) | 92.3 ± 8.0  (66.0, 99.0) | 89.7 ± 11.7  (38.8, 100) | 80.0 ± 14.9  (39.2, 95.5) |
|  | **LMCA** | 96.7 ± 8.1  (62.5, 100) | 96.7 ± 8.1  (62.5, 100) | 93.7 ± 15.8  (54.7, 100) | 84.0 ± 15.0  (50.0, 100) | 93.2 ± 13.6  (56.0, 100) | 80.8 ± 15.9  (42.9, 99.8) |
|  | **LCX** | 96.2 ± 5.2  (78.8, 100) | 94.1 ± 7.9  (68.1, 100) | 95.4 ± 6.3  (40.5, 100) | 83.1 ± 11.1  (52.7, 99.0) | 90.8 ± 14.3  (23.0, 100) | 75.3 ± 15.0  (31.2, 94.0) |

1. *McKenzie A, Van Herk M, Mijnheer B. Margins for geometric uncertainty around organs at risk in radiotherapy. Radiotherapy and Oncology. 2002;62(3):299-307.*
