# Supplementary material for: A modality‐agnostic coronary artery habitat model for cardiac sparing in radiotherapy
Source: Med Phys. 2026 Jul 21;53(8):e70595. doi: 10.1002/mp.70595 (PMC13389350; doi:10.1002/mp.70595)
Supplement: Supplementary file 8 — Supplementary Information [file MP-53-0-s012.docx]

Supplementary Table 6: Full Dosimetric Results for Re-optimized Plans

| **PATIENT 1** | | | | | | |
| --- | --- | --- | --- | --- | --- | --- |
| **ORGAN AT RISK (OAR)** | **WH-Spared Plan** | | **CA-Spared Plan** | | **Habitat-Spared Plan** | |
|  | D_0.03cc_ (Gy) | D_mean_ (Gy) | D_0.03cc_ (Gy) | D_mean_ (Gy) | D_0.03cc_ (Gy) | D_mean_ (Gy) |
| RCA | 19.6 | 11.1 | 10.8 | 8.3 | 8.9 | 6.8 |
| LADA | 33.3 | 17.3 | 15.3 | 10.5 | 14.0 | 9.5 |
| LMCA | 34.2 | 34.3 | 18.7 | 18.3 | 16.9 | 16.9 |
| LCX | 35.6 | 35.9 | 23.5 | 22.5 | 24.4 | 22.7 |
| Full hRCA | 32.5 | 10.5 | 43.6 | 11.5 | 27.9 | 8.3 |
| Full hLADA | 47.3 | 18.2 | 43.9 | 15.5 | 41.8 | 13.7 |
| Full hLMCA | 47.3 | 35.0 | 48.4 | 23.5 | 41.9 | 20.4 |
| Full hLCX | 46.9 | 28.4 | 56.1 | 28.6 | 51.3 | 28.3 |
| **PATIENT 2** | | | | | | |
| RCA | 27.2 | 14.5 | 14.7 | 9.8 | 11.5 | 7.2 |
| LADA | 31.4 | 11.0 | 12.6 | 7.0 | 10.3 | 6.0 |
| LMCA | 27.2 | 32.4 | 18.7 | 15.3 | 18.6 | 13.9 |
| LCX | 23.7 | 9.4 | 14.8 | 7.9 | 12.4 | 7.7 |
| Full hRCA | 45.8 | 12.5 | 47.2 | 10.0 | 38.4 | 7.0 |
| Full hLADA | 47.4 | 7.5 | 37.6 | 6.1 | 26.4 | 5.2 |
| Full hLMCA | 55.2 | 29.8 | 52.3 | 18.4 | 44.7 | 14.6 |
| Full hLCX | 51.1 | 9.2 | 38.9 | 8.0 | 29.9 | 7.4 |
| **PATIENT 3** | | | | | | |
| RCA | 23.1 | 14.9 | 19.2 | 15.7 | 19.3 | 14.4 |
| LADA | 28.6 | 18.9 | 19.6 | 14.7 | 12.5 | 9.3 |
| LMCA | 31.7 | 30.9 | 19.1 | 18.6 | 16.4 | 15.7 |
| LCX | 32.9 | 20.4 | 21.2 | 15.8 | 15.6 | 14.6 |
| Full hRCA | 31.6 | 10.9 | 42.3 | 14.3 | 34.9 | 12.6 |
| Full hLADA | 38.1 | 13.2 | 36.6 | 12.2 | 20.6 | 9.2 |
| Full hLMCA | 46.7 | 30.8 | 48.6 | 24.0 | 46.1 | 17.7 |
| Full hLCX | 40.3 | 14.4 | 35.5 | 13.5 | 25.2 | 12.3 |

hCA = habitat for coronary artery CA
