# Supplementary material for: A modality‐agnostic coronary artery habitat model for cardiac sparing in radiotherapy
Source: Med Phys. 2026 Jul 21;53(8):e70595. doi: 10.1002/mp.70595 (PMC13389350; doi:10.1002/mp.70595)
Supplement: Supplementary file 9 — Supplementary Information [file MP-53-0-s002.docx]

Supplementary Table 7: Dosimetric Comparison Between Re-optimized Plans

| **Patient 1** | | | | |
| --- | --- | --- | --- | --- |
| **ORGAN AT RISK (OAR)** | **Δ(WH – Habitat)** | | **Δ(CA – Habitat)** | |
|  | D_0.03cc_ (Gy) | D_mean_ (Gy) | D_0.03cc_ (Gy) | D_mean_ (Gy) |
| RCA | 10.7 | 4.3 | 1.9 | 1.5 |
| LADA | 19.3 | 7.8 | 1.3 | 1.0 |
| LMCA | 17.3 | 17.4 | 1.8 | 1.4 |
| LCX | 11.2 | 13.2 | -0.9 | -0.2 |
| Full hRCA | 4.6 | 2.2 | 15.7 | 3.2 |
| Full hLADA | 5.5 | 4.5 | 2.1 | 1.8 |
| Full hLMCA | 5.4 | 14.6 | 6.5 | 3.1 |
| Full hLCX | -4.4 | 0.1 | 4.8 | 0.3 |
| **Patient 2** | | | | |
| RCA | 15.7 | 7.3 | 3.2 | 2.6 |
| LADA | 21.1 | 5.0 | 2.3 | 1.0 |
| LMCA | 8.6 | 18.5 | 0.1 | 1.4 |
| LCX | 11.3 | 1.7 | 2.4 | 0.2 |
| Full hRCA | 7.4 | 5.5 | 8.8 | 3.0 |
| Full hLADA | 21 | 2.3 | 11.2 | 0.9 |
| Full hLMCA | 10.5 | 15.2 | 7.6 | 3.8 |
| Full hLCX | 21.2 | 1.8 | 9.0 | 0.6 |
| **Patient 3** | | | | |
| RCA | 3.8 | 0.5 | -0.1 | 1.3 |
| LADA | 16.1 | 9.6 | 7.1 | 5.4 |
| LMCA | 15.3 | 15.2 | 2.7 | 2.9 |
| LCX | 17.3 | 5.8 | 5.6 | 1.2 |
| Full hRCA | -3.3 | -1.7 | 7.4 | 1.7 |
| Full hLADA | 17.5 | 4.0 | 16 | 3.0 |
| Full hLMCA | 0.6 | 13.1 | 2.5 | 6.3 |
| Full hLCX | 15.1 | 2.1 | 10.3 | 1.2 |
