# Supplementary material for: A modality‐agnostic coronary artery habitat model for cardiac sparing in radiotherapy
Source: Med Phys. 2026 Jul 21;53(8):e70595. doi: 10.1002/mp.70595 (PMC13389350; doi:10.1002/mp.70595)
Supplement: Supplementary file 10 — Supplementary Information [file MP-53-0-s011.docx]

Supplementary Table 8: ICC Values with 95% Confidence Intervals for D_0.03cc_ and D_mean_ Between Main-Branch Habitats and Ground-Truth CAs

| Coronary Artery | D_mean_ | D_0.03cc_ |
| --- | --- | --- |
| RCA | 0.945 [0.904,0.968] | 0.874 [0.721,0.937] |
| LADA | 0.976 [0.959, 0.986] | 0.894 [0.753, 0.948] |
| LMCA | 0.961 [0.934, 0.978] | 0.855 [0.538, 0.939] |
| LCX | 0.981 [0.963, 0.989] | 0.918 [0.804, 0.960] |
| All Coronary Arteries | 0.970 [0.961, 0.977] | 0.887 [0.742, 0.940] |
