# Supplementary material for: A modality‐agnostic coronary artery habitat model for cardiac sparing in radiotherapy
Source: Med Phys. 2026 Jul 21;53(8):e70595. doi: 10.1002/mp.70595 (PMC13389350; doi:10.1002/mp.70595)
Supplement: Supplementary file 11 — Supplementary Information [file MP-53-0-s006.docx]

Supplementary Table 9: P-values from statistical testing between modalities, with p<0.05 marked with an asterisk, as determined via Mann-Whitney U test.

| Coronary Artery | Habitat Size | | | CA-Habitat Inclusion | | | Hausdorff Distance | | |
| --- | --- | --- | --- | --- | --- | --- | --- | --- | --- |
|  | CCTA-CT | CT-MR | CCTA-MR | CCTA-CT | CCTA-CT | CT-MR | CCTA-MR | CT-MR | CCTA-CT |
| RCA | 0.000* | 0.403 | 0.000* | 0.133 | 0.751 | 0.070 | 0.011* | 0.884 | 0.008* |
| LADA | 0.000* | 0.002* | 0.299 | 0.484 | 0.020* | 0.020* | 0.583 | 0.028* | 0.030* |
| LMCA | 0.000* | 0.232 | 0.000* | 0.172 | 0.102 | 0.363 | 0.245 | 0.079 | 0.303 |
| LCX | 0.000* | 0.377 | 0.001* | 0.986 | 0.329 | 0.219* | 0.874 | 0.533 | 0.759 |
