# Supplementary material for: A modality‐agnostic coronary artery habitat model for cardiac sparing in radiotherapy
Source: Med Phys. 2026 Jul 21;53(8):e70595. doi: 10.1002/mp.70595 (PMC13389350; doi:10.1002/mp.70595)
Supplement: Supplementary file 12 — Supplementary Information [file MP-53-0-s008.docx]

Supplementary Table 10: P-values from statistical testing between main and full-branch habitats on CCTA, with p<0.05 marked with an asterisk, as determined via Wilcoxon signed-rank test. Note that the main and full-branch LMCA definitions are the same, so its comparison has been excluded.

| Coronary Artery | Habitat Size | CA-Habitat Inclusion | Hausdorff Distance |
| --- | --- | --- | --- |
| RCA | 0.000* | 0.000* | 0.000* |
| LADA | 0.000* | 0.001* | 0.000* |
| LCX | 0.000* | 0.154 | 0.027* |
